# Pro/Anti-Inflammatory Cytokine Imbalance in Postischemic Left Ventricular Remodeling

**DOI:** 10.1155/2010/974694

**Published:** 2010-05-09

**Authors:** Pasqui Anna Laura, Di Renzo Michela, Maffei Silvia, Pastorelli Marcello, Pompella Gerarda, Auteri Alberto, Puccetti Luca

**Affiliations:** ^1^Division of Internal Medicine, Department of Clinical Medicine and Immunology, University of Siena, V. le Bracci, 53100 Siena, Italy; ^2^Department of Cardiology, University of Siena, V. le Bracci, 53100 Siena, Italy

## Abstract

*Objectives*. Cytokines play an important role in left ventricular remodeling consequent to myocardial ischemia. The aim of this study was to correlate cytokine production and lymphocyte apoptosis to post-ischemic left ventricular remodeling in patients affected by acute myocardial infarction (AMI) undergoing primary cutaneous angioplasty (PCI). *Methods*. In 40 patients, affected by AMI and undergoing PCI, we evaluated peripheral blood mononuclear cells (PBMCs), tumor necrosis factor-alpha (TNF-*α*) and interleukin 10 (IL10) production and apoptosis on day 1, day 3, day 7, 1 month and 6 months after PCI. Patients were divided into two subgroups of remodeling or not remodeling by echocardiographic criteria. *Results*. In the subgroup of remodeling patients, at each timepoint TNF-*α* production was increased significantly in comparison with the subgroup of not remodeling patients. IL10 production was statistically lower in remodeling subjects than in not remodeling ones 1 and 6 months after reperfusion. There were no differences between the two groups as regards lymphomonocyte apoptosis. *Conclusions*. We found an increased production of pro-inflammatory cytokine TNF-*α* and a corresponding decrease of anti-inflammatory/regulatory cytokine IL10 in remodeling patients and we concluded that this cytokine imbalance resulted in pro-inflammatory effects which might contribute to the progression of left ventricular remodeling.

## 1. Introduction

Inflammatory response and cytokine elaboration are integral components of the host immune response to tissue injury and play an active role after myocardial infarction. The degree of the inflammatory response is an important determinant of the host's outcome because it can lead to healing and restoration of function, to acute cardiac rupture, or chronic dilatation [1]. 

Myocardial remodeling can be defined as molecular, cellular, and interstitial changes within the myocardium that result in changes in left ventricular size and function [2, 3]: one of the predominant features of myocardial remodeling is left ventricular dilatation [4], whose progression affects the survival of the patients with an increased mortality due to heart failure [5]. 

Upregulation of pro-inflammatory cytokines, such as TNF*α*, interleukin 1beta (IL1*β*), and IL6, usually unexpressed in the normal heart, has been clearly shown both in animal and human models during myocardial infarction [6–8]; the acute intramyocardial gene expression of TNF*α*, IL1*β*, and IL6 involves both the infarct area and the noninfarcted myocardium, but it begins to decrease towards baseline after 1 week [9]. If the infarct size is large or if there are ongoing myocardial stress factors, the cytokine gene expression may remain elevated or may recrudescence as a second wave of cytokine activation [10]; the levels of late cytokine activation in the noninfarctual region correlate the best with the eventual left ventricular end-diastolic diameter measured at 20 weeks after infarction [1, 11]. More recently, it has been shown that IL10, a pleiotropic cytokine with potent anti-inflammatory effects in many cells lines [12], may have in some in vitro ischemia-reperfusion models a protective role through the suppression of the acute inflammatory process: in particular IL10 is able to abrogate TNF*α*-induced vascular smooth muscular cell (VSMC) growth [13].

On the basis of these premises, we studied the role of peripheral cytokine production in myocardial remodeling and functioning in patients affected by acute myocardial infarction undergoing PCI. In particular, we evaluated PBMCs TNF*α* and IL10 production as well as lymphomonocyte apoptosis at different timepoints after the acute ischemic event and we correlated immune activation to postischemic left ventricular remodeling assessed with echocardiographic study.

## 2. Materials and Methods

### 2.1. Patients

We enrolled 40 patients (mean age 64 years, range 48–78) affected by acute ST-elevation myocardial infarction (STEMI) undergoing PCI within 12 hours from the onset of symptoms. Patients were successively divided in two groups of left ventricular remodeling (R, *n* = 20) and no left ventricular remodeling patients (NR, *n* = 20) according the presence or absence of left ventricular dilation evaluated at 6 months after infarction with echocardiographic criteria (Tables 1 and 2). Patients with a history of previous myocardial infarction, previously known coronary artery disease, heart failure, arrhythmias, valvulopathies, chronic or acute inflammatory or infectious disease, neoplasm, liver, and renal disease were excluded from the study. The same conditions, particularly acute or chronic infections, induced the exclusion of patient from the study in the follow-up at the various timepoints. Patients did not assume drugs that could interfere with immune cell functions in any phase of the study; only acetaminophen was occasionally assumed by some patients (3 remodeling patients and 4 not remodeling subjects) and just for no longer than a few days and not in coincidence with blood sampling. Fifteen normal subjects matched for age and sex were also studied as regards to cell cultures. All patients gave their informed consent to be included in the study. The procedure was approved by the ethical committee of our Hospital and the study was done according the ethical standards for experiments in human subjects established by the Declaration of Helsinki.

### 2.2. Echodoppler Ultrasound Study

The study was done using Vivid seven echocardiograph (General Electrics) equipped with 2.5 MHz transducers. Bidimensional images, echo-Doppler ultrasound, and color-Doppler ultrasound spectra were analyzed using a computerized system (Echo-Dat; Medicine Inc.software). The ejection fraction (EF), left ventricular end-systolic (LVESV) and end-diastolic volume (LVEDV), were recorded as indices of ventricular function. EF was calculated by the modified Simpson's method in apical four- and two-chamber views [14, 15].

Postischemic left ventricular remodeling was defined as a 20% increase of left ventricular end-diastolic and end-systolic volumes at the end of follow-up (6 months) compared with baseline measures (day 1) [16].

### 2.3. Cell Isolation and Culture

PBMCs were obtained from the patients' and controls' heparinized whole blood at the following timepoints: day 1, day 3, day 7, 1 month, and 6 months after reperfusion. PBMCs were isolated by density gradient centrifugation over Histopaque 1077 (Sigma-Aldrich, Milano, Italy) and cultured in RPMI 1640 medium supplemented with fetal calf serum (10%), L-glutamine (2 mM), and penicillin-streptomycin (100 U/mL, 100 *μ*g/mL, Gibco, Paisley, UK).

### 2.4. TNF*α* and IL10 Production by PBMC

1 × 10^6^ PBMCs were put in culture in complete medium and stimulated with LPS (E.coli, 1 *μ*g/mL, Sigma) for 24 hours.

### 2.5. Quantification of TNF*α* and IL10

Cell culture media harvested were tested for TNF*α* and IL10 production by ELISA using ELISA microtiter plates (Corning Easy Wash, Celbio, Milano, Italy) coated overnight with 2 *μ*g/mL anti-TNF-*α* or anti-IL10 capture mAb (Pharmingen) in 0.1 M Na2HPO4 pH 9 buffer and blocked with PBS/Tween. A biotin-labeled anti-TNF*α* or anti-IL10 detecting antibody (Pharmingen) at 1 *μ*g/mL in PBS/10% fetal calf serum was used. The plates were developed using avidin-HRP (Vector, Burlingame, CA, USA) and 2,2 azino-bis substrate (Sigma). The lower limit of detection was 15.6 pg/mL.

### 2.6. Apoptosis Assays

After 20 hours of culture, lymphocyte apoptosis was assessed through the binding of Annexin V/uptake of propidium iodide by flow cytometry (PAS, Partec, Germany) using the apoptosis detection kit annexin V-FITC (Dako, Glostrup, Denmark). Forward light scatter characteristics of living cells were used to delete debris from the analyses. Logarithmic fluorescence intensity of annexin V-FITC was plotted versus the fluorescence intensity of propidium iodide (PI) in a dot plot. Data from 10.000 lymphocytes gated on forward scatter versus side scatter properties were analysed for each plot and the percentage of annexin positive- and PI negative-cells (early apoptotic cells) was determined. Quadrants were estimated by comparison of unstained and annexin V-FITC/PI stained samples.

### 2.7. Statistical Analysis

Data are presented as median ± standard error of the mean. The Mann Whitney *U*-test was used to compare data obtained in patients undergoing ventricular remodeling to those obtained in patients not undergoing ventricular remodeling and in controls. The Wilcoxon test was used to compare data obtained in the same group of patients at different timepoints. *P* < .05 was considered significant. Spearman's correlation coefficient was employed to determine putative linear relations between changes in the measurable echographic and biological parameters.

## 3. Results

### 3.1. Left Ventricular Remodeling

Table 1 shows baseline and clinical characteristics of patients divided in the two groups of patients who showed left ventricular remodeling, compared with patients who not undergo left ventricular remodeling. As shown in the table, the two subgroups of patients were very similar and no differences were present as regards risk factors, characteristics of infarction, and drug assumption. Table 2 shows the echocardiographic characteristics of the same groups of patients which were used to distinguish remodeling and not remodeling subjects on the basis of left ventricular dilation, defined as an increase in end diastolic volume >20%, as assessed by authors [16].

### 3.2. Patients Undergoing Left Ventricular Remodeling Showed an Increased Production of TNF*α*

**Figure 1:**
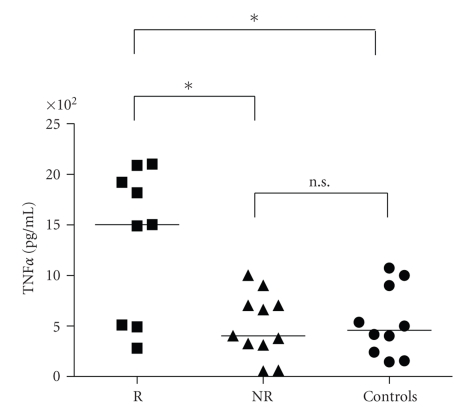
In patients developing left ventricular remodeling after myocardial infarction (R, *n* = 20) lymphomonocyte production of TNF-*α* in response to LPS was increased in comparison with patients not developing left ventricular remodeling (NR, *n* = 20) and in comparison with controls on day 1 after reperfusion (*P < .05).

**Figure 2:**
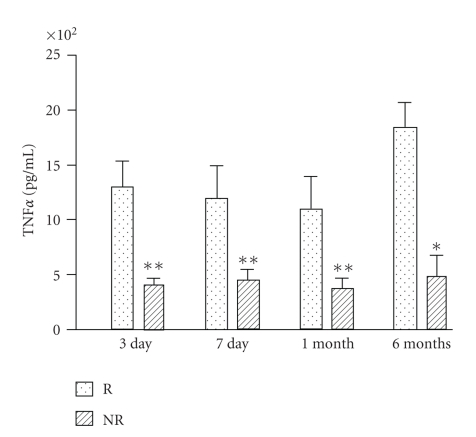
In patients developing left ventricular remodeling after myocardial infarction (R, *n* = 20) lymphomonocyte production of TNF-*α* in response to LPS was increased in comparison with patients not developing left ventricular remodeling (NR, *n* = 20) on day 3, on day 7, 1 month, and 6 months after reperfusion (*P < .05, **P < .01).

Patients with postischemic left ventricular remodeling showed on day 1 after PCI an increased production of TNF*α* after LPS stimulation in comparison with patients who did not undergo postischemic left ventricular remodeling (*P* < .05). They also produced increased amount of TNF*α* in comparison with controls (*P* < .05), whereas there was no difference between controls and patients who did not undergo left ventricular remodeling (Figure 1). The increased TNF*α* production after LPS stimulation was still detectable in remodeling subjects in comparison with not remodeling ones on day 3 (*P* < .01) and day 7 (*P* < .01) after PCI, as well as 1 month (*P* < .01) and 6 months (*P* < .05) after this procedure (Figure 2).

**Figure 3:**
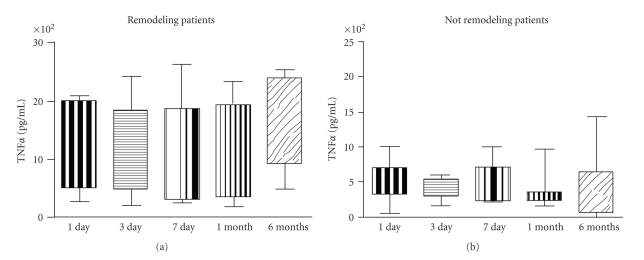
Both in patients developing left ventricular remodeling after myocardial infarction (*n* = 20) and in patients not developing left ventricular remodeling (*n* = 20) lymphomonocyte production of TNF-*α* in response to LPS was not different amongst the different timepoints evaluated.

In patients undergoing left ventricular remodeling we could not find any significant difference concerning LPS-stimulated TNF*α* production amongst the different timepoints; similar results were obtained in those patients who did not develop left ventricular remodeling (Figure 3).

### 3.3. Patients Undergoing Left Ventricular Remodeling Showed a Decreased Production of IL10

**Figure 4:**
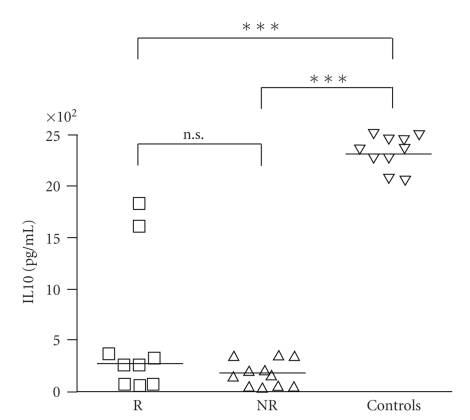
Both in patients developing left ventricular remodeling after myocardial infarction (R, *n* = 20) and in patients not developing left ventricular remodeling (NR, *n* = 20) lymphomonocyte production of IL10 in response to LPS was reduced in comparison with controls on day 1 after reperfusion (***P < .001).

**Figure 5:**
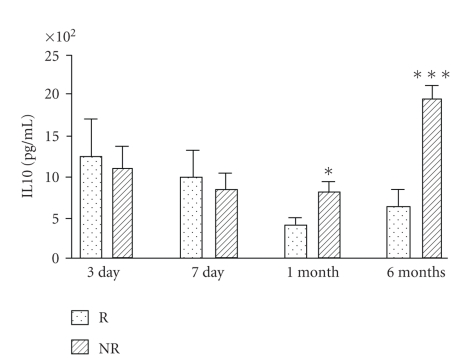
In patients developing left ventricular remodeling after myocardial infarction (R, *n* = 20) lymphomonocyte production of IL10 in response to LPS was reduced in comparison with patients not developing left ventricular remodeling (NR, *n* = 20) 1 month and 6 months after reperfusion (*P < .05 and ***P < .001, resp.).

When we analyzed LPS-induced IL10 production, on day 1 after reperfusion we did not find any difference between left ventricular remodeling subjects and not remodeling subjects. However in both groups IL10 production was significantly reduced in comparison with controls (*P* < .001, Figure 4). On day 3 and day 7 IL10 production was not different between remodeling and not remodeling subjects, but 1 and 6 months after reperfusion, IL10 production was statistically lower in subjects developing left ventricular remodeling than in not remodeling ones (*P* < .05, *P* < .001, Figure 5).

**Figure 6:**
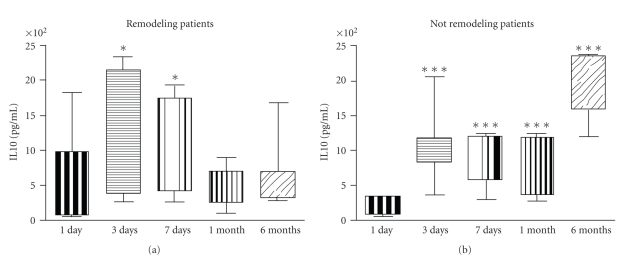
In patients developing left ventricular remodeling after myocardial infarction (*n* = 20) lymphomonocyte production of IL10 in response to LPS was increased on day 3 and day 7 after reperfusion in comparison with day 1 (*P < .05). In patients not developing left ventricular remodeling after myocardial infarction (*n* = 20) lymphomonocyte production of IL10 in response to LPS was increased on day 3, on day 7, 1 month, and 6 months after reperfusion in comparison with day 1 (***P < .001).

In patients undergoing left ventricular remodeling we found that LPS-stimulated IL10 production increased on day 3 and on day 7 in comparison with day 1 (*P* < .05) but returned to levels similar to day 1, 1 and 6 months after reperfusion (Figure 6). On the contrary, in not remodeling patients, IL10 production was significantly increased in comparison with day 1 after reperfusion at every timepoint (*P* < .001) and it was still statistically higher 6 months after reperfusion when it reached values comparable to normal controls (Figure 6).

### 3.4. Lymphocyte Apoptosis Was Not Increased in Patients Undergoing Left Ventricular Remodeling

When we analyzed spontaneous lymphocyte apoptosis in vitro after 24 hours of cultures, we found that the percentage of annexinV+PI- lymphocytes was not significantly different between remodeling patients and not remodeling ones (28.5 ± 10 versus 30 ± 8%), overlapping the values obtained in normal subjects (25 ± 11%) (not shown).

### 3.5. Linear Regression Analysis in the Whole Cohort

**Figure 7:**
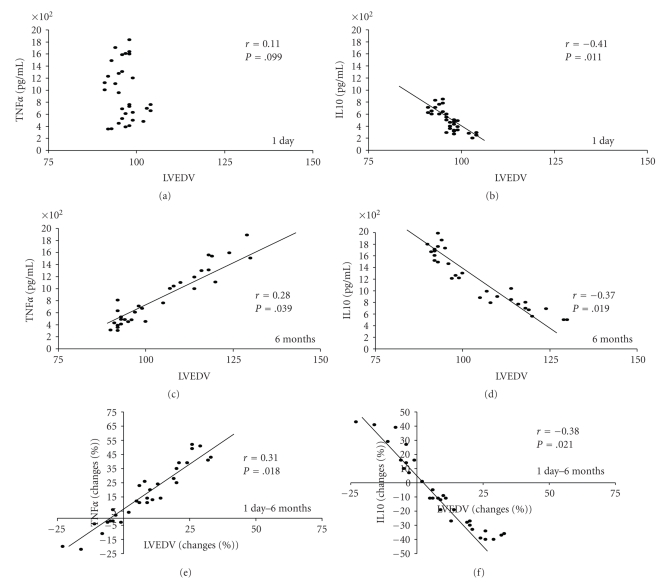
Linear regression coefficient (Spearman's) between TNF*α*, IL10, and Echocardiographic Left Ventricular end-diastolic volume (LVEDV) in the whole cohort (*n* = 40) at day 1, 6 months, and trend day 1–6 months. Each data is provided as continuous variable (day 1 and 6 months) and percent changes (trend day 1–6 months).

Our findings of a different pro-inflammatory/anti-inflammatory imbalance in the studied groups according to remodeling trend were confirmed by the linear regression analysis between TNF*α*, IL10, and LVEDV in the whole cohort (Figure 7). Indeed, a direct linear relation was found between TNF*α* levels and LVEDV volume (*r* = 0.28, *P* = .039) and between percent changes of both parameters (*r* = 0.31, *P* = .018) at six months (Figure 7). IL10 levels were inversely related to LVEDV at day 1 (*r* = −0.41, *P* = .011) that was confirmed at 6 months (*r* = −0.37, *P* = .019) also when analyzed as percent changes (*r* = −0.38, *P* = .021) (Figure 7).

## 4. Discussion

Postischemic left ventricular remodeling is a complex process that takes place after myocardial infarction both in the region affected by acute ischemia and in adjacent areas: it is characterized by reduction of myocytes in the infarct area, chamber dilatation, fibrosis and scar formation, collagen dissolution and excessive interstitial matrix accumulation, increased wall stress, and myocyte hypertrophy [17]. It results in ventricular geometric alterations and sustained deterioration in cardiac function [4, 5]. The development of ventricular remodeling involves a number of different mechanisms, including neurohormonal stimulation, enzymes, ion channels, oxidative stress, mechanical stress, growth factors, and inflammatory cytokine production [1]. 

Recent clinical and experimental studies have suggested that, among inflammatory cytokines, increased release of TNF*α* can contribute to the progression of left ventricular remodeling and dysfunction [18]. In adult rats, surgically implanted with an osmotic minipump infusing TNF*α* for 5 days in order to reach plasma levels comparable to those reported in clinical heart failure, a decrease in left ventricular fractional shortening occurred and left ventricular end diastolic diameter increased by over 25% when compared with time-matched controls [19]. In a transgenic mouse model, myocardial TNF*α* overexpression caused increased systolic and diastolic left ventricular volumes and a reduced collagen crosslinking in myocardium [20]. Since activation of TNF*α* receptors increases production of matrix metalloproteinases, one mechanism by which TNF*α* can influence left ventricular remodeling appears to be induction of myocardial matrix metalloproteinases and subsequent degradation of extracellular matrix components [20]. 

We found that in patients undergoing postischemic left ventricular remodeling in vitro TNF*α* production immediately after the ischemic event was statistically increased in comparison with patients who did not develop left ventricular remodeling. More interestingly, 6 months after myocardial infarction, in the same group of patients, TNF*α* production was persistently higher than that in the patients not undergoing left ventricular remodeling. This finding agrees with other authors studies which assessed the role of TNF*α* both in myocardial infarction and in left ventricular remodeling; particularly, it has been found that the levels of late cytokine activation in the noninfarctual region correlate the best with the eventual left ventricular end-diastolic diameter measured at 20 weeks after infarction [7, 8]. Our data suggest that peripheral cytokine production reflects what happens in myocardium in which the persistence of cytokine activation in response to mechanical, oxidative and neurohormonal stress initiates a vicious cycle. In patients undergoing ventricular remodeling secondary to ischemic cardiac disease, Otero et al. could not find increased serum levels of TNF*α* in comparison with those affected by dilated cardiomyopathy, whereas they found increased levels of sTNF-RI and sTNF-RII [21]; the different results obtained in this study in comparison with ours could be due to the fact that we evaluated TNF*α* in vitro production which does not necessary correspond to increased serum levels of this cytokine.

We also studied IL10 in vitro production because IL10 is an anti-inflammatory cytokine which modulates matrix metalloproteinase expression and suppresses pro-inflammatory cytokine synthesis, such as TNF*α* [12, 22]. Numerous recent experimental studies have shown that either systemic or local IL10 gene transfer attenuates atherogenesis and affects processes associated with lesion progression [23, 24]. Moreover, in a large cohort of patients affected by acute coronary syndromes, elevated IL10 serum levels have been associated with a more favourable prognosis [25]. However, at least in animal models, IL10 does not seem to play a critical role in infarct healing and left ventricular remodeling, because IL10 gene disruption does not alter fibrous tissue deposition and dilatative remodeling of the infarcted heart in a mouse model [26]. Similarly, the pretreatment of cardiac fibroblasts with IL10 does not inhibit chemokine synthesis in response to TNF*α* [26]. In our study, IL10 production was significantly decreased in comparison with normal subjects in both patients undergoing left ventricular remodeling and those not undergoing left ventricular remodeling, immediately after myocardial infarction. However, in those patients who did not develop left ventricular remodeling IL10 increased progressively reaching normal levels 6 months after the acute event, whereas in those developing left ventricular remodeling it increased on day 3 and 7 after reperfusion but returned to baseline levels 1 and 6 months after reperfusion. Therefore IL10 seems to play a protective role because lower levels several months after reperfusion are associated with left ventricular remodeling. Such findings could be of relevance because they add data to the body of evidence dealing with a more complex system in vivo, in which the balance between pro-/anti-inflammatory mediators is operating in cardiac remodeling with respect to the single action of several pro-/anti-inflammatory mediators as shown especially in animal models [26].

Finally we evaluated lymphocyte apoptosis because we found a persistent and increased production of TNF*α* in ventricular remodeling patients. TNF*α* is able to induce apoptosis in several cell types, including lymphocytes, by binding to TNFR-1 that contains a death domain [27, 28]. Moreover, in some human models, such as in aging, both TNF*α* production and TNF*α*-induced apoptosis are increased [29, 30]. However, spontaneous lymphocyte apoptosis was not different among ventricular remodeling patients, not ventricular remodeling ones and controls. It remains to be established whether the increased systemic TNF*α* production contributes to the increased cardiomyocyte apoptosis that has been found in postinfarction cardiac remodeling, especially at the site of recent infarction but also in unaffected areas to a lesser extent [31, 32].

In summary, our data analysing the cytokine network in the complex processes of postischemic left ventricular remodeling have confirmed important disturbances in the cell production of pro- and anti-inflammatory mediators; in particular the increase of pro-inflammatory cytokine TNF*α* is associated with the decrease of IL10 which could exert a protective/regulatory role. It is not clear why the same ischemic stress induces in patients with similar characteristics more TNF*α* and less IL10 production than in others. However we can observe that a marked imbalance between pro-inflammatory and protective mechanism is generated in patients undergoing left ventricular remodeling and we can suggest that the lack of this important homeostatic mechanism could influence the activation and the extent of inflammatory reaction which leads to the progression of ventricular remodeling and consequent myocardial failure.

## Figures and Tables

**Table 1:** Baseline characteristics of patients.

	Remodeling patients (*n* = 20)	Not remodeling patients (*n* = 20)	*P*
Age, y	64 ± 7	63 ± 9	NS
Male : female	13/7	12/8	NS
Risk factors, *n* (%)			
Familiarity	12 (60%)	10 (50%)	NS
Smoke	4 (20%)	6 (30%)	NS
Hypertension	2 (10%)	3(15%)	NS
Diabetes	0	0	NS
Hypercolesterolemia	4 (20%)	4 (20%)	NS
Anterior and/or lateral infarction	14 (70%)	13 (65%)	NS
Q wave at admission	12 (60%)	10 (50%)	NS
Time to reperfusion (hour)	5 ± 4	5 ± 3	NS
Troponin peak	4 ± 2	3.5 ± 1	NS
Treatment at discharge			
Antiaggregants	20 (100%)	20 (100%)	NS
B-blockers	12 (60%)	10 (50%)	NS
ACE-inhibitors	20 (100%)	20 (100%)	NS
Diuretics	16 (80%)	15 (75%)	NS
Nitrates	12 (60%)	12 (60%)	NS
Statins	20 (100%)	20 (100%)	NS
Baseline echocardiographic features			
LVEDV (mL)	93 ± 7	97 ± 6	NS
LVESV (mL)	53 ± 7	56 ± 7	NS
EF (%)	44 ± 4	42 ± 5	NS

LVEDV: left ventricular end-diastolic volume; LVESV: left ventricular end-systolic volume; EF: ejection fraction. NS: not significant; **P < .01.

**Table 2:** Echocardiographic evaluation.

	Pts with left ventricular remodeling			Pts without left ventricular remodeling		
	Day 1	6 months	Percent variation	Day 1	6 months	Percent variation

LVEDV (mL)	93 ± 7	128 ± 9	+38% ± 7	97 ± 6	93 ± 7	−4% ± 2
LVESV (mL)	53 ± 7	75 ± 7	+44% ± 7	56 ± 7	45 ± 6	−21% ± 3
EF (%)	44 ± 4	42 ± 4	−5% ± 2	42 ± 5	52 ± 4	+25 % ± 6

LVEDV: left ventricular end-diastolic volume; LVESV: left ventricular end-systolic volume; EF: ejection fraction.
