# Erratum to “Pro/Anti-Inflammatory Cytokine Imbalance in Postischemic Left Ventricular Remodeling”

**DOI:** 10.1155/2010/723589

**Published:** 2011-01-24

**Authors:** Anna Laura Pasqui, Michela Di Renzo, Silvia Maffei, Marcello Pastorelli, Gerarda Pompella, Alberto Auteri, Luca Puccetti

**Affiliations:** ^1^Division of Internal Medicine, Department of Clinical Medicine and Immunology, University of Siena, V. le Bracci, 53100 Siena, Italy; ^2^Department of Cardiology, University of Siena, V. le Bracci, 53100 Siena, Italy

The authors would like to inform the correct names for the present paper as stated above. 

